# Palliative Treatment of Esophageal Cancer Using Calcium Electroporation

**DOI:** 10.3390/cancers14215283

**Published:** 2022-10-27

**Authors:** Charlotte Egeland, Lene Baeksgaard, Julie Gehl, Ismail Gögenur, Michael Patrick Achiam

**Affiliations:** 1 Department of Surgery and Transplantation, Copenhagen University Hospital Rigshospitalet, 2100 Copenhagen, Denmark; 2 Department of Clinical Medicine, Faculty of Health and Medical Sciences, University of Copenhagen, 2200 Copenhagen, Denmark; 3 Department of Oncology, Copenhagen University Hospital Rigshospitalet, 2100 Copenhagen, Denmark; 4 Department of Clinical Oncology and Palliative Care, Center for Experimental Drug and Gene Electrotransfer (C*EDGE), Zealand University Hospital, 4000 Roskilde, Denmark; 5 Department of Surgery, Center for Surgical Science, Zealand University Hospital, 4600 Køge, Denmark

**Keywords:** esophageal cancer, calcium electroporation, malignant dysphagia, palliation

## Abstract

**Simple Summary:**

Calcium electroporation is a new cancer therapy wherein a high, rapid influx of calcium, facilitated by electrical pulses, is used to kill cancer cells. This pilot study aimed to evaluate the safety and feasibility of this new treatment for patients with non-curable esophageal cancer. The treatment was administrated during an endoscopic examination, under general anesthesia, and in an outpatient setting. Eight patients were treated. One severe adverse event occurred (requiring a single blood transfusion) and another three mild side effects were seen. Two patients reported dysphagia relief after treatment and one patient had a partial response evaluated by CT. Six months after treatment, the same patient was still in good condition, without the need for further treatment. Calcium electroporation was conducted in eight patients with only a few side effects. More studies are warranted to evaluate clinical efficacy.

**Abstract:**

Calcium electroporation (CaEP) is a novel cancer therapy wherein high intracellular calcium levels, facilitated by reversible electroporation, trigger tumor necrosis. This study aimed to establish safety with CaEP within esophageal cancer. Patients with non-curable esophageal cancer were included at Copenhagen University Hospital Rigshospitalet in 2021 and 2022. In an outpatient setting, calcium gluconate was injected intratumorally followed by reversible electroporation applied with an endoscopic electrode. The primary endpoint was the prevalence of adverse events, followed by palliation of dysphagia. All patients were evaluated with CT and upper endoscopies up to two months after treatment. The trial was registered at ClinicalTrials.gov (NCT04958044). Eight patients were treated. One serious adverse event (anemia, requiring a single blood transfusion) and three adverse events (mild retrosternal pain (two) and oral thrush (one)) were registered. Initially, six patients suffered from dysphagia: two reported dysphagia relief and four reported no change. From the imaging evaluation, one patient had a partial response, three patients had no response, and four patients had progression. Six months after treatment, the patient who responded well was still in good condition and without the need for further oncological treatment. CaEP was conducted in eight patients with only a few side effects. This study opens the way for larger studies evaluating tumor regression and symptom palliation.

## 1. Introduction

Esophageal cancer is the seventh most common cancer worldwide and the sixth leading cause of cancer death [1]. When diagnosed, less than 50% of patients can be offered potential curative treatment and the five-year survival is around 20% [2]. Palliative care consists of chemotherapy or radiotherapy [3], and if the patient suffers from severe dysphagia, an esophageal stent can be placed. However, several studies, including one from our institution, indicate that placing a stent might even worsen the survival outcomes [4,5,6]. Hence, new treatment options are warranted in this vulnerable patient group.

Our institution has previously published results from a first-in-man study wherein electrochemotherapy (ECT) was evaluated in patients with non-curable esophageal cancer [7]. Reversible electroporation is electrical pulses delivered directly to the cells. These pulses cause permeabilization of the cell membrane, which can further be combined with the administration of chemotherapeutic drugs [8]. Recently published studies and case reports have evaluated calcium electroporation (CaEP) as an alternative treatment to ECT in different malignant and premalignant tumors [9,10,11,12,13,14]. Our group performed a study wherein CaEP was evaluated in patients with Barret’s esophagus with high-grade dysplasia (preliminary data, ongoing data analysis). Furthermore, ongoing clinical trials are evaluating its role in colorectal cancer.

Human cells are sensitive to a high intracellular concentration of calcium, and when malignant cells are exposed to a rapid influx of calcium, tumor necrosis is induced [15]. The combination of intratumoral injection of calcium and locally applied high-frequency electrical pulses is termed CaEP [15]. As calcium is a much cheaper drug that is easier to handle and administrate, and, according to the available data, leads to fewer side effects (compared with chemotherapeutic drugs), several potential clinical benefits of CaEP exist. As no studies on CaEP in the upper gastrointestinal tract have been performed, this study aimed to establish the safety and feasibility of endoscopic-assisted CaEP in patients suffering from non-curable esophageal cancer.

## 2. Materials and Methods

This was a phase I, sponsor-investigator-initiated, observational, prospective, non-comparative trial of endoscopic-assisted CaEP in patients with non-curable esophageal cancer. The primary endpoint was the assessment of safety, evaluated by registration of adverse events (AEs) and serious adverse events (SAEs) occurring within the first 14 days after treatment. All events were categorized according to the Common Terminology Criteria for Adverse Events (CTCAE) scale, version 5.0 [16]. The treatment was intended as a one-time treatment when standard treatment had been offered first. If requested by the patient, and deemed suitable and appropriate by the investigators, the treatment could be repeated during the trial period.

The trial intended to treat eight patients but allowed for the inclusion of ten patients in case of dropouts. Inclusion criteria were as follows: age ≥ 18 years old, malignant tumor in the esophagus considered unsuitable for potential curative treatment, performance status ECOG/WHO ≤ 2 [17], expected survival > three months, platelet count > 50 billion/l, International Normalized Ratio (INR) < 1.5, willing and able to comply with the study procedures, women of childbearing potential (WOCBP) and male partners to WOCBP using adequate contraception, and written consent. Furthermore, all patients should have been offered or considered for standard oncological treatment before enrollment in the trial. Exclusion criteria were as follows: incorrigible coagulation disorder, a clinically significant cardiac arrhythmia, pregnancy or lactation, concurrent treatment with another investigational medicinal product, obstructive tumor of which the endoscope, with the attached electrode, could not pass (assessed during index endoscopy), and any other clinical condition or prior therapy that, in the opinion of the investigator, would make the patient unsuitable for the study or unable to comply with the study requirements.

**Figure 1:**
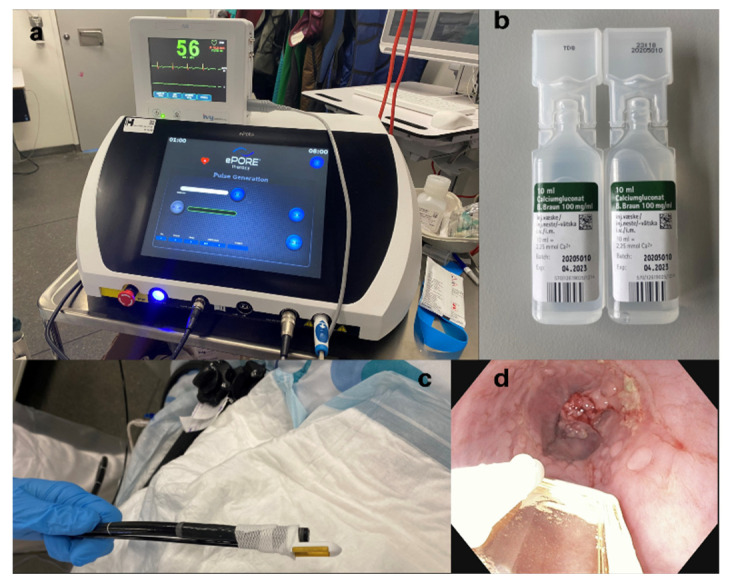
(**a**) The electroporator (ePORE^®^) is connected with a heart synchronization system. (**b**) Calcium gluconate. (**c**) The electrode (EndoVE^®^) is attached to the tip of the endoscope. (**d**) Calcium gluconate is injected into the tumor and the pulses are delivered. The procedure is repeated until the whole tumor area is covered.

Patients were recruited at the Department of Surgery and Transplantation and the Department of Oncology at Copenhagen University Hospital Rigshospitalet, Denmark. All procedures were performed in an outpatient setting under general anesthesia (propofol and remifentanil) by an upper GI surgeon with great experience in upper GI endoscopy. Before the procedure, antibiotics (metronidazole (1000 mg) and piperacillin/tazobactam (4 g/0.5 g)) and dexamethasone (16 mg) were administrated intravenously. When the patient was anesthetized, calcium gluconate (B.Braun, Melsungen, Germany, 0.23 mmol/mL) was injected intratumorally. Calcium gluconate was chosen, as we had previously observed that after injection with calcium chloride, delivering the pulses could be a problem due to increased electrical impedance in the tissue. The dosage varied depending on tumor size and length, and a maximum dosage of 20 mL was allowed. The drug was injected with a needle into several parts of the tumor, approximately 2 mL at every spot. The exact dosage was not calculated beforehand but administered into the mucosa until it was assessed that the whole tumor area was covered. Thereafter, the electrode was attached to the scope. In this trial, EndoVE^®^ (Mirai Medical, Galway, Ireland) was used. The electrode consists of a chamber and two parallel electrodes, and when connected with a gastroscope (or colonoscope) it allows for the delivery of electrical pulses. The electrode was connected to a pulse generator, ePORE^®^ (Mirai Medical, Ireland), a surgical suction pump, and an ECG trigger monitor (Figure 1). Hereafter, the electrical pulses were delivered to the tumor area. High-frequency bipolar pulses of 5000 kHz and with a voltage of 1000 V were used. With the ECG synchronization, each pulse was delivered in approximately 20 s. The pulses were repeated until the whole tumor area was covered. After extubating, the patient was observed in the postoperative ward and was discharged the same afternoon.

Follow-up included upper endoscopies performed within the first week and 2–4 weeks postoperatively and a thoracic/abdominal CT scan performed at 2–4 weeks and 6–8 weeks, respectively. The response was categorized as complete response (no visual tumor in the treated area), partial response, stable disease, or local progression. Furthermore, palliation of pain (evaluated by numeric rating scale (NRS)) and dysphagia (evaluated by Mellow Pinka scale [18]), as well as evaluation of quality of life (QoL) (EORTCQLQ-C30), were assessed. The 90-day survival was registered for all participants.

To ensure a realistic and implementable subject number, the study size was determined to be a maximum of ten participants. Demographics and procedure details are described individually for all patients. Due to the low number of participants, only descriptive statistics were used to describe and present AEs and SAEs, as well as the results regarding palliation and QoL. Missing data were analyzed for a pattern of missingness. The statistical analysis was conducted in R, version 3.6.1 (5 July 2019) [19].

The protocol was approved by the Danish Medicines Agency (EudraCT no.: 2020-005787-58), the Regional Ethics Committee (H-20082119), and the Regional Department of Research and Innovation. The trial was monitored by the Good Clinical Practice Unit at Copenhagen University Hospital, Denmark, and registered at ClinicalTrials.gov (NCT04958044) before the first patient was enrolled.

## 3. Results

### 3.1. Demographics

The trial was conducted from June 2021 to May 2022. Ten patients were included (eight men and two women, aged 57–83 years). Eight out of the patients were treated, and one patient was treated twice. Two male patients were excluded prior to treatment. In one case, exclusion was due to disease progression and rapidly decreased performance status, and in the other case, severe lung disease made the patient unsuitable for general anesthesia. All patients had previously received oncological treatment due to their current cancer disease. Time since diagnosis varied from 6 to 39 months (mean time: 19 months). All general characteristics and previous oncological history are described in Table 1.

### 3.2. Safety Evaluation

During the procedures, no intraoperative complications occurred. In five out of nine procedures, no adverse events were reported within the first 14 days. Two patients reported mild retrosternal pain (grade 1) the first days after treatment, one patient reported worsening anemia (grade 3), and one patient developed oral thrush (grade 2). Out of these events, one was considered a serious adverse event (one-day hospitalization due to anemia, requiring a single blood transfusion). The patient who was treated twice was scheduled for a third treatment; however, the procedure was canceled in the endoscopic suite as the patient had atrial fibrillation and the generator could not deliver the pulses with the ECG synchronization system. Thereafter, the patient’s condition rapidly worsened, which is why he was not scheduled for a new treatment session. Primary and secondary endpoints are described in detail in Table 2.

### 3.3. Palliation and Quality of Life

In up to eight weeks after treatment, pain, dysphagia, and QoL were regularly assessed and compared with baseline using questionnaires. Initially, six out of the eight treated patients suffered from dysphagia. During the follow-up period, two patients reported dysphagia relief, five reported no change, and one patient reported worsening dysphagia after two months (this patient did not suffer from dysphagia before treatment). Regarding pain palliation, three patients reported retrosternal pain before treatment. One patient reported pain relief, three patients reported unchanged symptom status, and three patients reported increased pain during the trial period (one patient was missing). Self-reported perception of general health status within the first month after treatment was unchanged in three patients, whereas three patients experienced an impaired general health status (two patients did not fill out the questionnaire before treatment). One patient reported that his/her general quality of life improved during the first months, four patients reported no change, and two patients reported impaired quality of life (one questionnaire was missing).

### 3.4. Imaging Response

**Figure 2:**
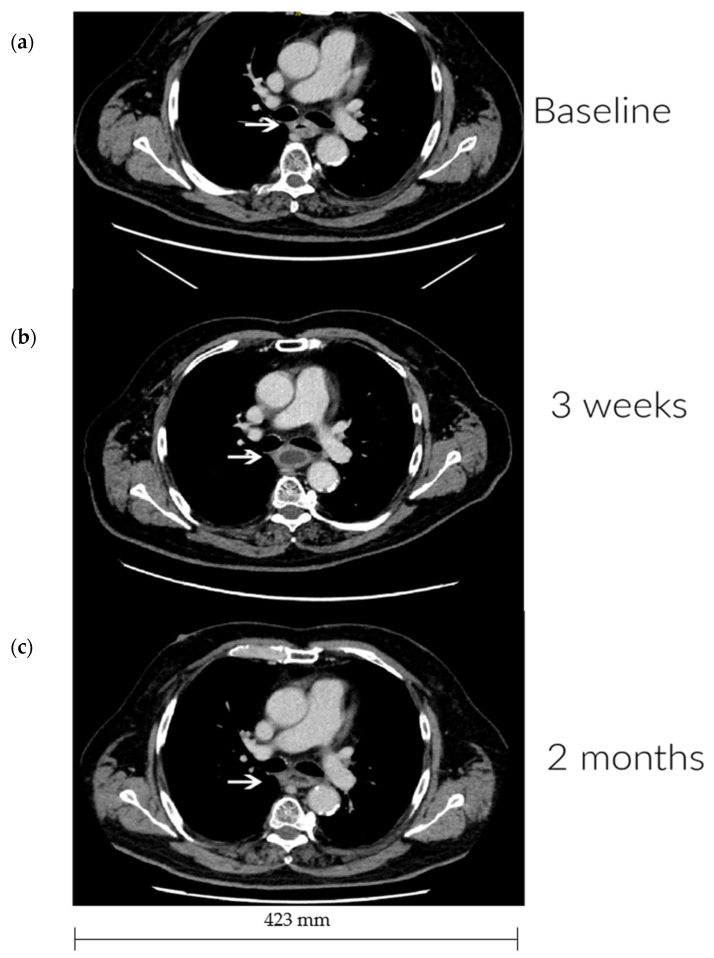
Imaging response. CT imaging at (**a**) baseline, (**b**) three weeks, and (**c**) two months after treatment. The arrows indicate the tumor localization in the thoracic part of the esophagus. (**a**) Largest esophageal wall thickness is 10 mm. A left main bronchial impression is seen due to tumor size. (**b**) Diminishing circumferential wall thickness corresponding to the treated area. The bronchial impression is no longer observed. (**c**) No further changes, stable disease.

All patients underwent a baseline CT scan and a minimum of one additional evaluation CT. From the evaluation CT, it was observed that one patient had a partial tumor response (Figure 2), three patients had stable disease, and four patients had tumor progression during the follow-up period. No response in distant metastases was seen.

### 3.5. Visual Tumor Response from Endoscopic Examinations

**Figure 3:**
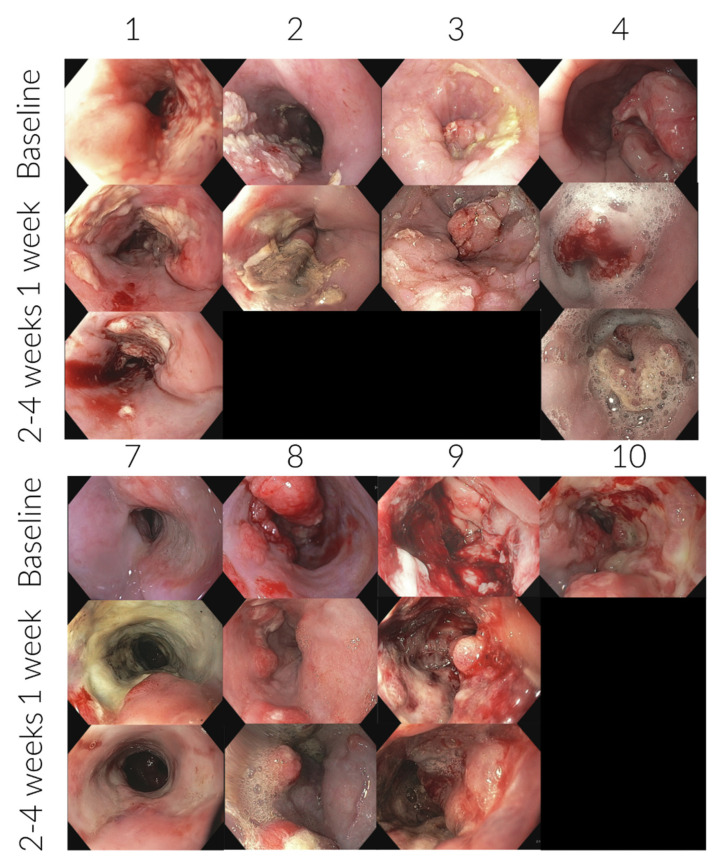
Endoscopic findings, patient ID 1-10 (only patients who received treatment). Endoscopic images from the tumor site before treatment and again at follow-up. It is clear to see that already within the first week, in most of the patients, the treated area is covered by a fibrin layer. In no patients was a total visual response seen.

Seven patients underwent an endoscopic examination within a week after treatment (the last patient was canceled due to discomfort during the procedure) and in six of these patients, the examination was repeated 2–4 weeks after treatment. Endoscopic images of the tumors and the treatment response is presented in Figure 3. In four cases, the treated mucosa was covered by a thin fibrin layer. A clear and sharp boundary between the untreated and treated tissue was seen shortly after treatment. In the patient who had a treatment response on CT, the fibrin-layered tissue was replaced by almost normal tissue after one month, ID 7. Six months after treatment, the patient was still in good condition and without the need for further oncological treatment. One patient (ID 8) had a long tumor segment wherein the oral parts of the tumor, consisting of several tumor islands, was primarily treated. These tumor islands were less extensive already after three days. However, no visual response in the anal, more obstructive part of the tumor was seen. In two patients, no visual response was seen (ID 3 and 9).

### 3.6. Survival

Seven of the eight treated patients were still alive 90 days after treatment, and one patient died two months after the given treatment due to disease progression.

## 4. Discussion

From this first-in-man pilot trial, it is concluded that endoscopic-assisted calcium electroporation is a safe procedure with few side effects. We found some signs of tumor response from both imaging acquisition and endoscopic examinations. However, due to the limited data, the palliative effect and tumor response are hard to evaluate and must be further investigated in larger clinical trials.

Up to today, only a few clinical trials have been published examining CaEP as a cancer treatment [9,10,12,13] and no CaEP trials have been published concerning esophageal cancer. Our research group performed a trial where patients with Barret’s esophagus with high-grade dysplasia were treated with CaEP before an endoscopic resection (unpublished data). In the current trial, one tumor responded to the given treatment (evaluated from CT imaging) and five of the patients had visual signs of response evaluated from endoscopic examinations. This is less than a previous study by Plaschke et al. [9], who found tumor response (evaluated according to RECIST criteria using magnetic-resonance imaging) in three out of six patients with recurrent head and neck squamous-cell carcinoma. Like our trial, all patients had already received standard treatment and were not candidates for other oncological or surgical treatment options when they were referred for the CaEP trial. Plaschke et al. applied the electrical field with a needle electrode under general anesthesia where it was possible to get a good overview of the whole tumor area. Even though some signs of response were seen from the endoscopic examinations in our patients, the lacking tumor response may partly be explained by the difficulties in applying the electric fields rather than the lack of efficiency from CaEP. CaEP has been proven efficient in both clinical and pre-clinical trials [9,10,12,13,20,21]. Another explanation could be that the pulses do not penetrate the tissue deeply enough when applied with this type of electrode (in often hard, stenotic tumor tissue). However, these are only speculations, as this study was small and mostly explorative. As the treatment was not very efficient, the tumor progression experienced during the follow-up period in four of the patients was predictable. The patients were already considered ineligible for other oncological treatments, and CaEP was the only treatment they received during the period. For patients in these advanced disease stages, the expected survival time is short.

In terms of dysphagia palliation, we did not see a very convincing response. For non-obstructive tumors, there are several treatment options, such as radiotherapy, argon plasma coagulation, and balloon dilatation. However, with these modalities, the effect of the treatment only lasts a very limited amount of time and the treatment must be repeated. For obstructive tumors, if radiotherapy is not efficient or already administered, placing an esophageal stent is the only option. As it has been shown that placing a stent might have a negative impact on survival time [4,5,6], other treatment options are warranted. As the electrode is designed today, the treatment cannot compete with an esophageal stent, as it cannot pass the tumor. However, if a long-lasting response (>2–3 months) from CaEP can be achieved in the esophagus, the treatment could potentially prolong the time to stent or simply avoid it if used in earlier stages of the disease. As with other volume-reduction therapies in the esophagus (i.e., radiotherapy), a great tumor response can potentially lead to perforation and even death, and this risk must be taken into account when determining the treatment course.

The electrode used in this trial, EndoVE^®^, has some limitations. First, patients with obstructive tumors cannot be treated at all and were not included in this trial. This leads to a selection bias where the patients who might suffer the most are rejected. Secondly, even if the endoscope and the attached electrode can pass by the tumor, navigation, maneuverability, and a good luminal view during treatment are challenging. It is not obvious where the pulses have been delivered and the risk of untreated areas is very real. However, if implementing the treatment in earlier tumor stages this shortcoming may not be present. In smaller, softer tumors, where the tumor tissue can be sucked into the chamber, the EndoVE^®^ probe is probably more suitable. In colorectal tumors, where intraluminal space is often larger, the electrode has been proven easier to use [22]. Recently, preliminary results have been published wherein three colorectal tumors were treated with ECT using a needle electrode with good results [23]. Theoretically, a needle electrode might also be possible in the esophagus. Ongoing trials are evaluating CaEP with this current electrode in colorectal cancer (ClinicalTrials.gov Identifier: NCT03694080, NCT03542214 (preliminary results are presented as an abstract), and NCT04816045).

The most used drug combined with electroporation is bleomycin. Treatment with bleomycin has known disadvantages and limitations, including well-described side effects, the process of mixing and handling the drug, and the fact that it cannot be readministered infinitely. By exploring and introducing calcium as a possible drug instead, some of these disadvantages could be overcome. It is cheaper, easier to handle, and when using calcium gluconate, it does not even have to be mixed. This is a huge advantage, especially in an endoscopy suite. Furthermore, it has a long shelf life and the treatment can be repeated. Besides ECT and CaEP, irreversible electroporation (IRE) has been explored as a potential cancer treatment within different clinical fields since it was first described in 2005 [24]. IRE is high-voltage electrical pulses applied to the tumor cells, leading to irreversible cell-membrane permeabilization and cell necrosis [25]. Even though IRE is predominately non-thermal, the treatment induces some degree of thermal damage in the ablation zone [26]. In the esophagus, with the proximity to the heart, the aorta, and the trachea, this must be carefully considered. Furthermore, when applying high-voltage pulses in organs close to the heart, even with heart synchronization, cardiac arrhythmias have been observed [27,28]. Hence, some clear advantages of using lower-voltage pulses combined with calcium (or chemotherapeutic drugs) do exist.

In this trial, we choose to use calcium gluconate instead of calcium chloride, which has been used in other clinical trials. In a previous trial (unpublished data) we discovered that after injecting calcium chloride into the esophageal tissue it could be hard to deliver the pulses due to higher electrical impedance in the mucosa. A lack of reduction in impedance after the pulses are delivered can be read out of the generator during the procedure. In addition, calcium gluconate has the advantage of not being needed to solute with saline, as the mixture has the correct concentration directly off the shelf. Regarding calcium gluconate volume, we chose a rather restrictive approach and set the maximum allowed volume to 20 mL (0.23 mmol/mL). Due to previous irradiation and hard tumors in many cases, injection led to spilling through the mucosa. In circumferential tumors extending over a longer area, it would probably be necessary to inject more calcium to ensure the whole tumor area is covered.

This area of research, to improve the treatment of esophageal cancer, is of high importance. The survival prognosis is poor, with an expected five-year survival of only 20%, with many of the patients already having distant metastases when diagnosed. In individual patient cases [29,30] and in preclinical studies with both ECT with bleomycin and CaEP [31,32,33,34] the treatment has been shown to induce a systemic immunological response. This is interesting, as CaEP and/or ECT may hold the potential of going from a local treatment only to a systemic treatment in the future. Even more interesting, it has been suggested that ECT, followed by an immunological treatment, might lead to an even more convincing tumor response [35]. In a single case, a patient with highly aggressive skin cancer was treated with ECT followed by one dose of Avelumab (anti-programmed death-ligand 1 (PD-L1) monoclonal antibody) and experienced an excellent treatment response [30]. It was suggested that ECT helped to establish a favorable microenvironment in the malignant cells, which further enhanced the effect of the Avelumab.

Conclusions from this exploratory study should be interpreted with caution due to the low patient number. Furthermore, due to the design of the electrode, not all patients were treated sufficiently. As calcium gluconate can be administered several times (in contrast to bleomycin), the treatment can potentially be repeated and the tumor stepwise debulked until the whole tumor area is covered.

## 5. Conclusions

In conclusion, CaEP was completed in eight patients with advanced esophageal cancer with no other available treatment options. The procedure was safe, no severe complication was seen, and there were only mild side effects. Larger studies must be conducted in order to evaluate tumor response and palliation.

## 6. Patents

Calcium electroporation is patented, which may result in financial gain for the co-inventor and investigator in this trial, Julie Gehl. The patent is managed by the act on inventions at public research institutions and the patents act in collaboration with the Office of Technology Transfer in the Capital Region of Denmark.

## Figures and Tables

**Table 1:** Baseline characteristics. Baseline characteristics from all ten patients, including demographics, tumor characteristics, previous oncological treatment, initial dysphagia score, and treatment data.

Patient ID	Gender/Age	Tumor Type/Location	Previous Treatment	Initial Dysphagia Score *	Treatment Data	Treatment Data Comments
1	Male/76 yr	Adc/Lower third	6 series (Carboplatin, Docetaxel, Capecitabine) RT: 3 Gy x 10 F	0	20 mL (0.23 mmol/L) calcium gluconate 7 pulses 20 min procedure time	Only the esophageal part of the tumor was treated, meaning tumor tissue in the stomach was not included in the treatment area.
2	Male/83 yr	Scc/Middle third (partly obstructive	dCRT: 2 Gy x 25 F + 5-Fluorouracil RT: 2Gy x 25 F (lymph node) + 5-Fluorouracil	1	1st treatment: 13 mL 12 pulses 46 min 2nd treatment: 5 mL 3 pulses 20 min	1st treatment: Balloon dilatation was necessary before intubation with EndoVE^®^. 2nd treatment: The electrode was detached several times and the endoscope needed to be retracted to place the electrode again. Only the oral part of the tumor was treated.
3	Male/59 yr	Adc/Lower third (obstructive)	6 series (Capecitabine, Oxaliplatin)	3	20 mL 7 pulses 35 min	Due to an obstructive tumor, only the oral part of the tumor was treated. 5 mL of calcium gluconate was spilled.
4	Male/62 yr	Adc/Middle and lower third	1 series (Capecitabine, Trastuzumab) RT: 3 Gy x 10 F (brain) RT: 8 Gy x 1 F (femur, tibia)	NA	No treatment	
5	Male/75 yr	Adc/Middle and lower third	4 series FLOT (Docetaxel, Oxaliplatin, 5- Fluorouracil, Leucovorin) dCRT: 2 Gy x 25 F + 5-Fluorouracil	1	No treatment	
6	Male/62 yr	Adc/Lower third	3 series (Capecitabine, Oxaliplatin + placebo/add-on (blinded randomized trial)) 6 series (Docetaxel) RT: 3 Gy x 10 F	2	19 mL 12 pulses 18 min	Uncomplicated procedure
7	Male/78 yr	Adc/Middle third (local recurrence after dCRT)	dCRT: 2 Gy x 25 F + 5-Fluorouracil	0	19 mL 13 pulses 15 min	Uncomplicated procedure
8	Female/66 yr	Adc/Middle and lower third	5 series (Capecitabine, Oxaliplatin) 1 series (Docetaxel)	3	19 mL 20 pulses 45 min	Uncomplicated procedure
9	Female/72 yr	Adc/Lower third	9 series (Capecitabine, Oxaliplatin, Trastuzumab) 12 series (Trastuzumab) 3 series (Docetaxel)	1	20 mL NA pulses 25 min	Poor overview with the electrode attached due to tumor localization involving the gastroesophageal junction.
10	Male/57 yr	Adc/Middle and lower third	9 series (Capecitabine) 3 series (Docetaxel) 2 series (Irinotecan) RT: 3 Gy x 10 F	1	20 mL 20 pulses 30 min	Uncomplicated procedure

Adc = adenocarcinoma; Scc = squamous-cell carcinoma; RT = radiotherapy; Gy = gray; F = fraction; dCRT = definitive chemoradio therapy, NA = Not Available. * 0 = able to swallow normal food/no dysphagia, 1 = able to swallow some solid food, 2 = able to swallow only semi-solid food, 3 = able to swallow only liquids [18].

**Table 2:** Primary and secondary endpoints, overview.

Patient ID	AEs (CTCAE Grade)	Dysphagia	Pain	Imaging Response
1	Worsening of anemia (3) *	0, 0, 0, 0	0, 0, 0, 0	Stable disease (1 month)
2	Retrosternal pain (1)	1, 1, 1, -	1, 3, 3, -	Stable disease (1 month)
3	-	3, 3, 3, -	7, 7, 0, -	Progression (1 month)
4	-	-	-	-
5	-	1, -	1, -	-
6	Retrosternal pain (1)	2, 2, 2, -	6, 8, 7, -	Progression (1 month)
7	-	0, 0, 0, 1	0, 0, 0, 0	Partial response (2 months)
8	Oral thrush (2)	3, 1, 1, 1	0, 0, 0, 0	Progression (1 month)
9	-	1, 0, 0, 0	0, 4, 1, 1	Progression (1 month)
10	-	1, 1, 1, -	-	Stable disease (1 month)

AEs = adverse events; CTCAE = Common Terminology Criteria for Adverse Events scale, version 5.0 [16]; dysphagia score: 0 = able to swallow normal food/no dysphagia, 1 = able to swallow some solid food, 2 = able to swallow only semi-solid food, 3 = able to swallow only liquids [18]; pain score: Numeric Rating Scale 0–10. AEs were reported within 14 days after treatment, pain and dysphagia scores were registered at baseline, within one week, at 2–4 weeks, and at 6–8 weeks after treatment, respectively. * Serious AE (one-day hospitalization, requiring a single blood transfusion).

## Data Availability

Raw data were generated at the Department of Surgery and Transplantation, University Hospital Rigshospitalet, Copenhagen, Denmark. Derived data supporting the findings of this study are available from the corresponding author (Charlotte Egeland) on request.
